# Loss of Fas apoptosis inhibitory molecule leads to spontaneous obesity and hepatosteatosis

**DOI:** 10.1038/cddis.2016.12

**Published:** 2016-02-11

**Authors:** J Huo, Y Ma, J-J Liu, Y S Ho, S Liu, L Y Soh, S Chen, S Xu, W Han, A Hong, S C Lim, K-P Lam

**Affiliations:** 1 Immunology Group, Bioprocessing Technology Institute, Agency for Science, Technology and Research, 20 Biopolis Way, #06-01 Centros, Singapore 138668, Singapore; 2 Institute of Biomedicine, Ji Nan University, 601 HUANG PO DA DAO XI, Guang Zhou 510632, P.R. China; 3 Clinical Research Unit, Khoo Teck Puat Hospital, ALEXANDRA HEALTH PTE LTD, 90 Yishun Central, Singapore 768828, Singapore; 4 Metabolomics Group, Bioprocessing Technology Institute, Agency for Science, Technology and Research, 20 Biopolis Way, #02-01 Centros, Singapore 138668, Singapore; 5 Laboratory of Metabolic Medicine, Singapore Bioimaging Consortium, Biomedical Sciences Institutes, 11 Biopolis Way, Helios, Singapore 138667, Singapore; 6 Diabetes Center, Khoo Teck Puat Hospital, ALEXANDRA HEALTH PTE LTD, 90 Yishun Central, Singapore 768828, Singapore; 7 Department of Physiology, National University of Singapore, NUS Yong Loo Lin School of Medicine, Block MD9, 2 Medical Drive #04-01, Singapore 117597, Singapore; 8 Department of Microbiology, National University of Singapore, 5 Science Drive 2, Blk MD4, Level 3, Singapore 117545, Singapore; 9 School of Biological Sciences, Nanyang Technological University, 60 Nanyang Drive, Singapore 637551, Singapore

## Abstract

Altered hepatic lipogenesis is associated with metabolic diseases such as obesity and hepatosteatosis. Insulin resistance and compensatory hyperinsulinaemia are key drivers of these metabolic imbalances. Fas apoptosis inhibitory molecule (FAIM), a ubiquitously expressed antiapoptotic protein, functions as a mediator of Akt signalling. Since Akt acts at a nodal point in insulin signalling, we hypothesize that FAIM may be involved in energy metabolism. In the current study, C57BL/6 wild-type (WT) and FAIM-knockout (FAIM-KO) male mice were fed with normal chow diet and body weight changes were monitored. Energy expenditure, substrate utilization and physical activities were analysed using a metabolic cage. Liver, pancreas and adipose tissue were subjected to histological examination. Serum glucose and insulin levels and lipid profiles were determined by biochemical assays. Changes in components of the insulin signalling pathway in FAIM-KO mice were examined by immunoblots. We found that FAIM-KO mice developed spontaneous non-hyperphagic obesity accompanied by hepatosteatosis, adipocyte hypertrophy, dyslipidaemia, hyperglycaemia and hyperinsulinaemia. In FAIM-KO liver, lipogenesis was elevated as indicated by increased fatty acid synthesis and SREBP-1 and SREBP-2 activation. Notably, protein expression of insulin receptor beta was markedly reduced in insulin target organs of FAIM-KO mice. Akt phosphorylation was also lower in FAIM-KO liver and adipose tissue as compared with WT controls. In addition, phosphorylation of insulin receptor substrate-1 and Akt2 in response to insulin treatment in isolated FAIM-KO hepatocytes was also markedly attenuated. Altogether, our data indicate that FAIM is a novel regulator of insulin signalling and plays an essential role in energy homoeostasis. These findings may shed light on the pathogenesis of obesity and hepatosteatosis.

Obesity and related metabolic diseases have reached epidemic level worldwide. Metabolic disorders including hyperglycaemia, dyslipidaemia, hypertension and central obesity often constellate as ‘metabolic syndrome'.^1, 2^ Insulin resistance and compensatory hyperinsulinaemia underline these metabolic imbalances.^3^ Hepatic insulin resistance may stem from compromised signalling through the insulin receptor, insulin receptor substrate (IRS) and other downstream effectors such as the Akt kinase.

Liver-specific insulin receptor knockout mice exhibited insulin resistance and glucose intolerance accompanied by hyperinsulinaemia.^4^ Thus intact hepatic insulin signalling is critical for glucose metabolism.^5^ Further studies indicated that hepatic insulin resistance also accounted for dyslipidaemia^6^ and steatosis.^7^

Hepatic insulin resistance is also associated with reduced expression of IRS-1 and IRS-2. Earlier studies showed that reduced IRS-1 expression in liver led to decreased glucokinase expression and increased blood glucose, whereas knockdown of IRS-2 resulted in elevated lipogenesis due to upregulation of SREBP-1c and fatty acid synthase and increased hepatic lipid accumulation. The dual knockdown of IRS-1 and IRS-2 resulted in systemic insulin resistance and hepatosteatosis.^8^

As a downstream serine/threonine kinase, Akt is a critical mediator of the metabolic actions of insulin.^9^ Akt2 is the predominant isoform in insulin-responsive tissues such as liver, muscle and adipose tissue.^10^ Impaired Akt signalling has been studied in livers of animal models of insulin resistance, such as *ob/ob* and lipodystrophic mice.^11, 12^ Liver-specific disruption of Akt2 has mild effects on glycaemia but more dramatic effects on lipogensis in those obese mice.^10, 13^

Fas apoptosis inhibitory molecule (FAIM) is a critical mediator of Akt activation^14, 15^ and was initially cloned from antigen-activated B cells and shown to protect them from activation-induced cell death.^16^ FAIM is ubiquitously expressed and conserved in evolution although it bears no homology to any known protein.^16^ Alternate splicing of FAIM generates a short (FAIM-S) and long (FAIM-L) isoform.^17^ FAIM-L is expressed in neurons.^18^ Studies demonstrate that FAIM-L contributes to inhibition of X-linked inhibitor of apoptosis protein (XIAP) ubiquitination and determines the protective or deleterious effect of tumor necrosis factor-*α* in neuronal cells.^19, 20^ As a widely expressed antiapoptotic molecule, FAIM-S has been shown to participate in nuclear factor kappa-light-chain-enhancer of activated B-cell activation and prolong the lifespan of neuronal cells in culture.^21^ Our group had previously generated mice deficient in FAIM and demonstrated that FAIM played a critical role in protecting developing thymocytes and hepatocytes from Fas killing.^14, 22^ Furthermore, we found FAIM expression to be highly expressed in symptomatic multiple myeloma patients compared with normal or premalignant individuals.^15^ Our earlier studies also showed that FAIM mediates Akt activation in thymocytes^14^ and multiple myeloma cells.^15^ Mechanistically, FAIM modulates the localization of Akt to lipid rafts during its activation.^14^ Since Akt acts as a nodal point in insulin signalling^23^ and maintains glucose and lipid homoeostasis,^10, 13^ it is conceivable that FAIM might play a role in insulin signalling and maintenance of metabolic homoeostasis.

The goal of the experiments reported herein is to demonstrate that antiapoptotic molecule FAIM is a novel regulator of energy homoeostasis and its expression is essential for the integrity of the insulin signalling pathway.

## Results

### FAIM-deficient mice develop non-hyperphagic obesity

**Figure 1:**
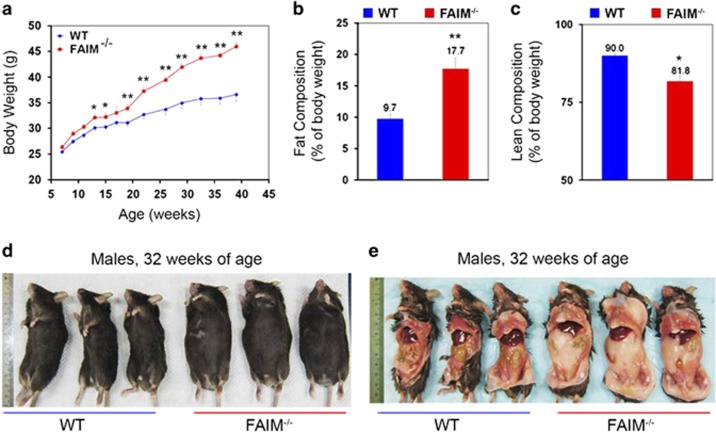
FAIM-deficient mice on normal chow diet develop obesity. (**a**) Body weight changes of wild-type (WT, *n*=16) and FAIM-deficient (FAIM^−/−^, *n*=21) male mice at 39 weeks of age on normal chow diet (NCD). Data are mean±S.E.M. (*P<0.05, **P<0.01 *versus* corresponding WT group). (**b** and **c**) EchoMRI analysis of Fat/lean body composition of WT and FAIM^−/−^ males (*n*=6, **P<0.01) at 14 weeks of age on NCD. (**d** and **e**) Representative photograph of ventral and exposed ventral view of WT and FAIM^−/−^ males at 32 weeks of age, showing fat accumulation in mutant mice

Wild-type (WT) and FAIM-deficient (FAIM-KO) male mice were fed with normal chow diet (NCD) (calories from protein, fat and carbohydrate were 24, 11 and 65%, respectively) for 39 weeks and changes in their body weight were recorded weekly. As shown in Figure 1a, body weight gain was faster in FAIM-KO as compared with WT controls. The body weight gain in FAIM-KO mice accelerated from 19 weeks of age, and by 32 weeks, they manifested an overt obese phenotype with ~30% body weight increase compared with WT controls (Figures 1a, d and e). Full body magnetic resonance imaging (MRI) revealed that FAIM-KO mice had significantly higher fat composition (Figure 1b) and lower lean mass (Figure 1c) as compared with WT controls at 14 weeks of age, that is, before the onset of overt weight gain.

**Figure 2:**
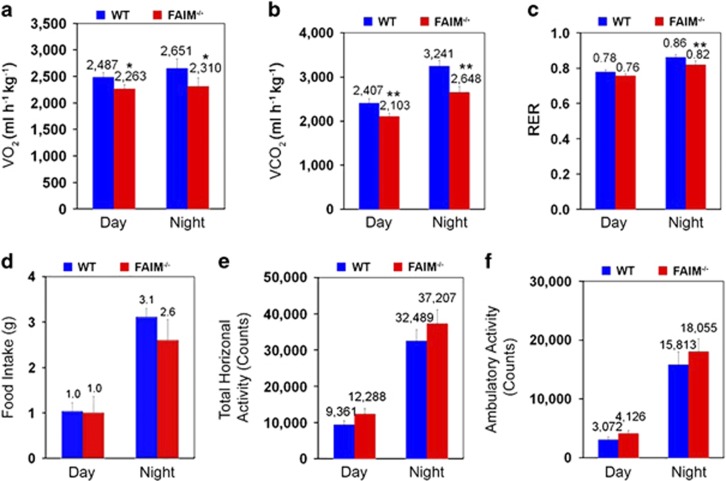
Lose of FAIM promotes non-hyperphagic obesity. Metabolic analyses of WT (*n*=6, BW=31.23±2.72 g) and FAIM^−/−^ males (*n*=6, BW=34.06±2.56 g, *P*=0.09) at 14 weeks of age on NCD. Oxygen (O_2_) consumption (**a**) and carbon dioxide (CO_2_) production (**b**) were measured over a night and day cycle. The respiratory exchange ratio (RER) was assessed during the day and night cycles (**c**). Food (**d**) intake in a day and night cycle for WT and FAIM^−/−^ mice was monitored. Physical activity including total horizontal activity (**e**) and total ambulatory activity (f) were depicted as the average total count per day or night cycle. Data are presented as mean±S.E.M. (*n*=6). *P<0.05, **P<0.01 *versus* the corresponding WT group

To characterize energy metabolism in FAIM-KO mice, we examined their energy expenditure, substrate utilization and physical activities using metabolic cage analyses. For meaningful comparisons, WT and FAIM-KO mice subjected to metabolic cage analysis were of 14 weeks of age and had similar body weight (31.23±1.72 g in WT *versus* 34.06±2.56 g in FAIM-KO mice, *P*=0.09). FAIM-KO mice exhibited significantly reduced oxygen consumption (Figure 2a), carbon dioxide production (Figure 2b) and respiratory exchange ratio (RER) at night time (Figure 2c). No significant difference in food intake (Figure 2d) was observed, suggesting that mutant mice have spontaneous non-hyperphagic obesity. Interestingly, no reduction in locomotor activities was observed during day and night time in mutant as compared with WT mice (Figures 2e and f). Taken together, these data suggested that reduction in resting metabolic rate, but not hyperphagia or physical inactivity, might account for the obesity seen in FAIM-deficient mice.

### FAIM-deficient mice develop hypertrophic adipocytes and hepatosteatosis

**Figure 3:**
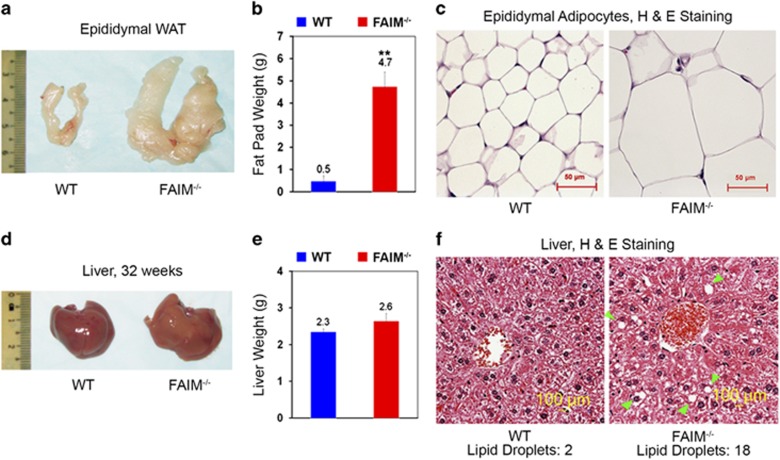
Obesity and hepatosteatosis in FAIM-deficient mice. (**a–c**) Gross examination, tissue weight and haematoxylin and eosin (H&E) staining of epididymal white adipose tissue (WAT) of WT and FAIM^−/−^ males at 32 weeks of age on NCD (*n*=3, **P<0.01). Scale bar, 50 *μ*m. (**d–f**) Gross outlook, tissue weight and H&E staining of liver tissue of WT and FAIM^−/−^ males at 32 weeks of age on NCD (*n*=4). Scale bar, 100 *μ*m. Green arrows point to lipid droplets. Numbers of lipid droplets were shown

To gain insight into the cause of obesity in FAIM-KO mice, we examine their fat tissue. The weight of the epididymal fat pad in FAIM-KO mice was increased ~8.4-fold (Figures 3a and b) and the size of their adipocytes was markedly enlarged (Figure 3c) as compared with WT controls. The mutant liver looked pale and slightly enlarged (Figure 3d) but there was no significant difference in the gross weight compared with WT liver. However, haematoxylin and eosin staining showed more lipid droplet accumulation in mutant livers (Figure 3f), indicating that FAIM-KO mice developed hepatic steatosis.

### FAIM deficiency enhances sterol regulatory element-binding protein (SREBP) signalling and promotes lipogenesis in liver

**Figure 4:**
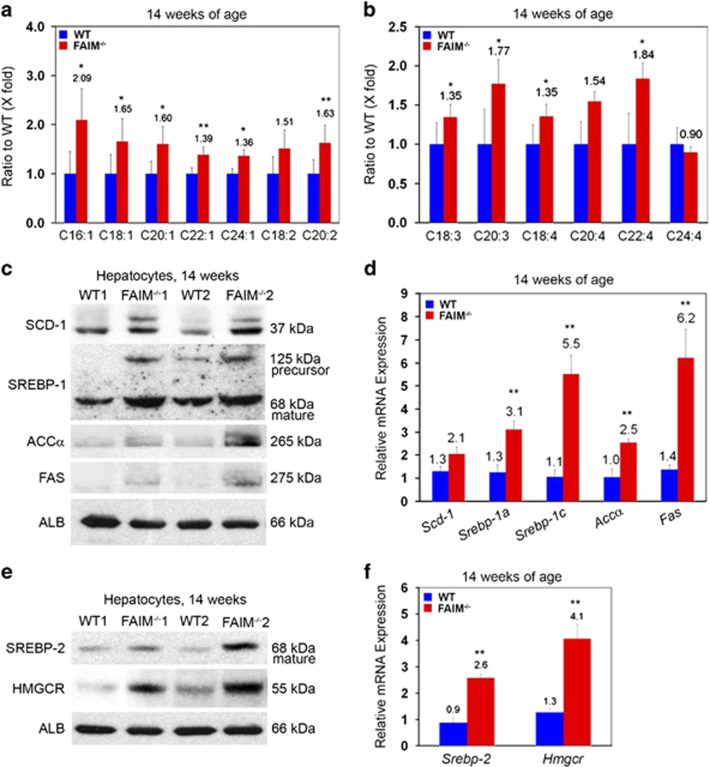
Elevated hepatic lipogenesis and SREBP signalling in FAIM-deficient mice. Metabolomics analysis of the ratio of hepatic unsaturated (**a** and **b**) fatty acids in WT and FAIM^−/−^ males (*n*=3) fed with NCD at 14 weeks of age. Data are expressed as mean±S.E.M. *P<0.05, **P<0.01 *versus* the corresponding WT value. (**c** and **d**) Protein and mRNA expression of SCD-1, SREBP-1(125 kDa, precursor; 68 kDa, mature) ACC*α* and FAS in liver tissues of WT and FAIM^−/−^ males fed with NCD at 14 weeks of age (*n*=3, **P<0.01). (**e** and **f**) Protein and mRNA expression of SREBP-2 and HMGR in liver tissues of WT and FAIM^−/−^ males (*n*=3, **P<0.01). WT and FAIM^−/−^ mice were 14 weeks of age on NCD

Liquid chromatography-mass spectrometry (LC-MS)-based lipid analysis was performed to characterize fatty acid metabolism in WT and FAIM-KO livers. At 14 weeks of age, that is, before the onset of overt weight gain, saturated fatty acid [C16:0] was significantly increased in the livers of FAIM-KO mice as compared with WT controls (Supplementary Figure S1). Notably, monounsaturated fatty acids ([C16:1], [C18:1], [C20:1], [C22:1] and [C24:1]) and polyunsaturated fatty acids ([C20:2], [C18:3], [C20:3], [C18:4] and [C22:4]) were markedly elevated (Figures 4a and b), suggesting increased lipogenesis in FAIM-KO mice. Concordantly, the expression of SREBP and its downstream lipogenic target genes such as stearoyl-CoA desaturase 1 (SCD-1),^24^ fatty acid synthase (FAS)^25, 26, 27^ and acetyl-CoA carboxylase^28^ were all markedly increased in hepatocytes isolated from FAIM-KO mice (Figure 4c) compared with control. Quantitative real-time PCR (qRT-PCR) analysis showed that gene expressions of *Srebp-1a, Srebp-1c, Accα* and *FAS* were significantly upregulated in the mutant liver (Figure 4d). Apart from the upregulation of SREBP-1a and SREBP-1c which preferentially enhances fatty acid synthesis, we also observed upregulation of the SREBP-2 pathway that preferentially activates cholesterol synthesis in the liver by targeting 3-hydroxy-3-methyl-glutaryl-CoA reductase (HMGCR) (Figures 4e and f).^29, 30, 31^

### FAIM-deficient mice exhibit dyslipidaemia, hyperglycaemia and hyperinsulinaemia

**Figure 5:**
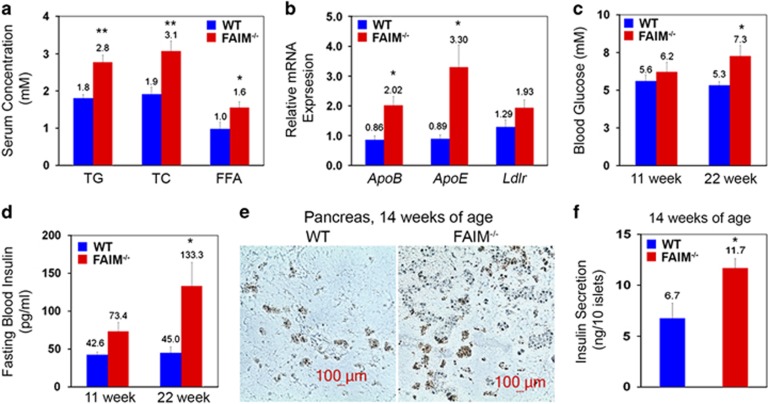
Dyslipidaemia, hyperglycaemia and hyperinsulinemia in FAIM-deficient mice. (**a**) Fasting serum concentrations of triglyceride (TG), total cholesterol (TC) and free fatty acid (FFA) in WT and FAIM^−/−^ males (*n*=3) fed with NCD at 17 weeks of age. (**b**) Relative mRNA expression of ApoB, ApoE and LDL receptor (Ldlr) in WT and FAIM^−/−^ males (*n*=3) fed with NCD at 17 weeks of age. Data are expressed as mean±S.E.M. (*n*=3) *P<0.05, **P<0.01 *versus* corresponding WT groups. Fasting serum glucose (**c**) and insulin (**d**) levels in WT and FAIM^−/−^ males fed with NCD at 17 weeks of age (*n*=3). (**e**) Representative immunohistochemical staining of insulin in the pancreas of WT and FAIM^−/−^ males (*n*=3) at 14 weeks of age. Scale bar, 100 *μ*m. (**f**) Insulin production in isolated islets from WT and FAIM^−/−^ males (*n*=3) at 14 weeks of age treated with glucose (11  mM) for 2 h in culture media

We next examined the blood lipid profile of FAIM-KO mice. As compared with WT controls, total sera cholesterol, triglycerides and free fatty acids levels were all significantly elevated in the mutants from 17 weeks of age onwards (Figure 5a). Consistently, *ApoB, ApoE* and *Ldlr* mRNA expressions were also elevated in FAIM-KO livers (Figure 5b).

Since hepatosteatosis, dyslipidaemia and hyperglycaemia often cluster and give rise to ‘metabolic syndrome',^32^ we next examined blood glucose levels in FAIM-KO mice. The fasting blood glucose level remained normal in FAIM-KO mice at 11 weeks of age (6.2±1.3 *versus* 5.6±0.9 mM in controls, *P*>0.05). However, they exhibited fasting hyperglycaemia at 22 weeks of age (7.2±1.4 *versus* 5.3±0.5 mM in controls, *P*<0.05) (Figure 5c). Further analysis by peritoneal glucose tolerance tests (GTTs) revealed that glucose disposal in skeletal muscle was normal in young (10 weeks) FAIM-KO mice whereas glucose intolerance occurred in older (50 weeks) mutants (Supplementary Figures S2a and b).

As hyperinsulinaemia underlies metabolic syndrome, we next examined fasting serum insulin levels in FAIM-KO and WT mice. Fasting serum insulin level was ~2-fold higher in mutant mice at 22 weeks of age as compared with WT controls (133.3±69.0 *versus* 45.0±17.9 pg/ml; Figure 5d). Additionally, immunohistochemical staining of insulin in pancreas revealed islet hyperplasia in FAIM-KO mice (Figure 5e). Furthermore, isolated islets from 14-week-old FAIM-KO mice displayed increased insulin secretion in response to high glucose (11 mM) stimulation *in vitro* (Figure 5f).

### FAIM deficiency diminishes insulin actions in insulin target tissues

**Figure 6:**
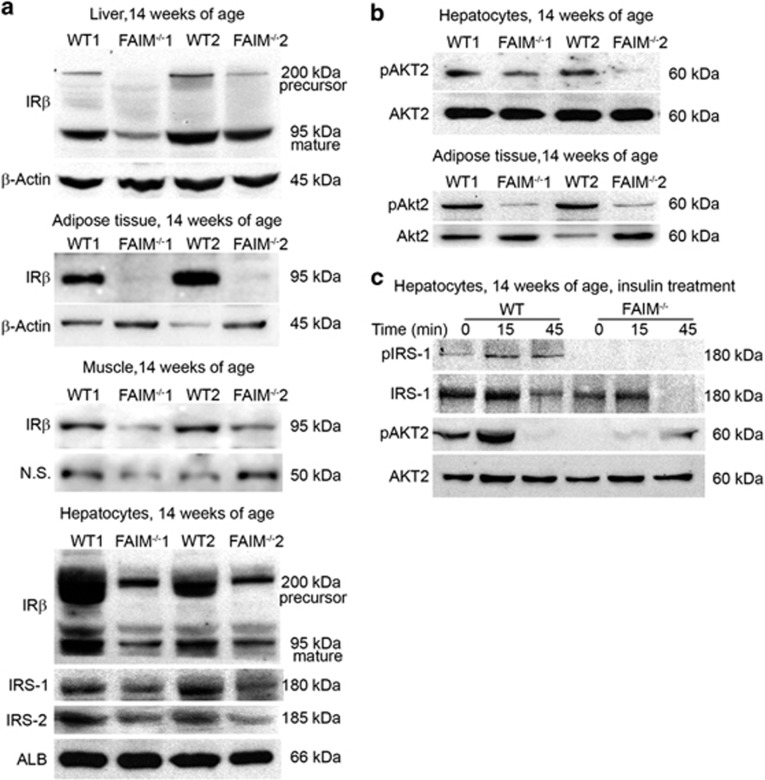
FAIM regulates insulin signalling and protein expressions of IR*β*,IRS-1 and IRS-2. (**a**) Protein expression of precursor IR*β* (~200 kDa) and mature IR*β* (~95 kDa) in liver tissues of WT and FAIM^−/−^ males at 14 weeks of age. IR*β* expression (~95 kDa) in fat tissue and muscle of WT and FAIM^−/−^ males at age of 14 weeks fed with NCD. N.S., nonspecific bands. Protein expression of precursor IR*β* (~200 kDa), mature IR*β* (~95 kDa), IRS-1 and IRS-2 in isolated hepatocytes of WT and FAIM^−/−^ males at 14 weeks of age (lower panel). (**b**) Phosphorylation (Ser474) of Akt2 in isolated hepatocytes (upper) and adipose tissue (lower) of WT and FAIM^−/−^ males fed with NCD at 14 weeks of age. (**c**) Insulin (50 mU/ml) induced IRS-1 (Ser612) and Akt2 (Ser474) phosphorylation in isolated WT and FAIM^−/−^ hepatocytes (representative of three western blots)

Hepatic insulin resistance is an important pathophysiological feature of hepatosteatosis and obesity. Hence, we attempted to elucidate the molecular mechanisms by which FAIM deficiency led to energy dysmetabolism. Insulin receptor, downstream IRS proteins and phosphatidylinositol-3-OH kinase (PI(3)K)/Akt are the main components of insulin signalling pathway.^33^ Marked reduction in insulin receptor beta (IR*β*) expression was observed in *ex vivo* mutant liver, adipose tissues and skeletal muscle. Protein levels of IR*β*, IRS-1 and IRS-2 were also significantly reduced in FAIM-KO hepatocytes as compared with WT controls (Figure 6a). Impaired Akt signalling and decreased IRS expression in the livers have been shown to be the underlying causes of insulin resistance in *ob/ob* and lipodystrophic mice.^11, 12^ Our earlier studies had shown that FAIM expression was essential for the activation of Akt in thymocytes^14^ and myeloma cells.^15^ Therefore, it is conceivable that FAIM deficiency may lead to impaired Akt activation and defective insulin signalling in insulin target tissues. Indeed, Akt2 phosphorylation (Ser474) was reduced in hepatocytes and adipose tissue (Figure 6b) isolated from FAIM-KO mice. Furthermore, Akt2 (Ser474) and IRS-1 (Ser612) phosphorylation in response to insulin treatment (50 mU/ml) were also attenuated in FAIM-KO hepatocytes compared with WT controls (Figure 6c). These findings reinforced the hypothesis that FAIM plays a role in regulating insulin signalling.

### FAIM expression may associate with metabolism dysregulation in human

The findings from the study of FAIM-deficient mice prompted us to examine the relevance of FAIM in human obesity and metabolic dysfunction. We analysed FAIM expression in peripheral leukocytes as they were more readily accessible than liver biopsies and had also been widely used in studies on insulin signalling and metabolic diseases.^34, 35, 36, 37^ We recruited 33 obese subjects (19 females and 14 males, age 53±18 years old, body mass index (BMI) 30.9±4.4 kg/m^2^, fasting blood insulin 16.2±3.8 mU/l, HOMA-IR 5.1±1.9) and 14 lean controls (6 females and 8 males, age 26±4 years old, BMI 20.9±1.7 kg/m^2^, fasting blood insulin 9.2±1.2 mU/l, HOMA-IR 2.0±0.4) (Supplementary Figures S3a, c and d). FAIM expression in leukocytes was significantly lower in obese subjects as compared with lean controls (relative expression 0.57±0.25 *versus* 1.02±0.20, *P*<0.0001) (Supplementary Figure S3b). Bivariate correlation analysis showed that FAIM expression was inversely correlated with BMI, plasma insulin levels and HOMA-IR, an established indicator of insulin resistance (Supplementary Figures S4a–c). The multivariable linear regression model further revealed that BMI and HOMA-IR were independently associated with FAIM expression after adjusting for multiple potential confounders including age (Supplementary Table S1).

## Discussion

In our present study, we uncovered a novel role for FAIM as a metabolic regulator of insulin signalling, lipogenesis and metabolic homoeostasis. We found FAIM-KO mice to develop an obese phenotype even on NCD. The spontaneous non-hyperphagic obesity in FAIM-KO mice was accompanied by enhanced lipogenesis in the liver, hypertrophic adipocytes, dyslipidaemia, hyperglycaemia and hyperinsulinaemia. The phenotype of FAIM-KO mouse resembles human metabolic syndrome which is a constellation of dyslipidaemia, hyperglycaemia, hypertension and central obesity. These observations extend our previously work by showing that, in addition to its role as a regulator of apoptosis, FAIM is critically involved in maintaining energy metabolism.

Mechanistically, we found FAIM to be involved in insulin signalling. This is evidenced by reduced phosphorylation of Akt at Ser474 in both FAIM-KO liver and adipose tissues *ex vivo* and the weakened response (resistance) to insulin treatment in FAIM-KO hepatocytes *in vitro*. These observations are consistent with our earlier studies showing that FAIM regulates Akt activation in thymocytes and myeloma cells.^14, 15^ Therefore, the hyperinsulinaemia observed in FAIM-deficient mice might arise as compensation to insulin resistance. It also in part explains the enhanced activation of SREBP signalling and increased lipogenesis in FAIM-KO mice. Further examination of the insulin signalling pathway in FAIM-KO mouse revealed that FAIM likely regulates the expressions of IR*β*, IRS-1 and IRS-2. As shown in Figure 6a, the expressions of IR*β*, IRS-1 and IRS-2 were reduced in FAIM-KO hepatocytes and IR*β* protein expression was also markedly decreased in *ex vivo* mutant liver, adipose tissues, skeletal muscle and isolated hepatocytes. These findings extended our early finding by showing that FAIM may modulate Akt activation by regulating the expression of insulin receptor and IRS.

Interestingly, the insulin signalling defects in FAIM-KO mice were not concurrently accompanied by glucose intolerance as showed by the GTT (Supplementary Figure S2). This observation suggested that glucose disposal in skeletal muscle was not affected by the reduced insulin receptor and IRS expression in FAIM-KO mouse. This seemingly counterintuitive finding can be reconciled by earlier studies showing that glucose tolerance was normal in fat-specific and muscle-specific insulin receptor knockout mouse models.^38, 39^

It has been known that FAIM is a regulator of apoptosis. We have previously shown that FAIM-deficient mice have an apoptosis-related phenotype.^22^ Obesity in FAIM-KO mice may result from either hyperplasia (increase in adipocyte number) or hypertrophy (increase in size of adipocytes). We did not characterize apoptosis and proliferation in insulin target tissues such as adipose tissues. However, we did not observe overt cell death in liver, pancreas or fat tissues in this study. On the other hand, histological study showed that the adipocytes in FAIM-KO mice were markedly enlarged (Figure 3c). Therefore, the obese phenotype in FAIM-KO mice could be attributed to adipocyte hypertrophy instead of changes in adipocyte numbers. On the other hand, FAIM may potentially modulate inflammatory cytokine/chemokine production since it plays a role in cell death receptor signalling. Indeed, a recent paper has shown that FAIM modulates inflammation response in neuronal cells.^20^ Given the close relationship between chronic inflammation, insulin resistance and metabolic diseases, it is worthwhile to examine whether FAIM regulates insulin actions by modulating inflammatory pathways. This work is currently in progress.

As an evolutionally conserved and broadly expressed molecule, FAIM exists in two isoforms, with the long isoform (FAIM-L) expressed in neuronal tissues and short isoform (FAIM-S) ubiquitously expressed.^21^ In our mouse model, both FAIM-L and FAIM-S were deficient which prevented us from dissecting the isoform-specific function of FAIM-L and FAIM-S in the regulation of energy metabolism. This will be of interest in future studies.

Our current study may have clinical implications. Metabolic disease has become an epidemic worldwide. Insulin resistance and the associated hyperinsulinaemia are the main causes of energy dysmetabolism. Our preliminary clinical study showed that lower FAIM expression in leukocytes may be correlated with biomarkers of insulin resistance and obesity in humans. However, we cautioned that our study sample is small and there are inherent differences between lean and obese groups although we have attempted to address this issue by multivariable analysis to adjust for potential confounders such as age. On the other hand, although peripheral leukocyte has been widely used for the study of insulin signalling and metabolic disease,^34, 35, 36^ we are aware of the potential limitation of this methodology. Therefore, the clinical observation in our study can only be taken as hypothesis generating. A carefully planned large clinical study is warranted to examine the role of FAIM in energy metabolism in humans.

In summary, FAIM deficiency leads to phenotypic changes in mice resembling human metabolic syndrome. Mechanistically, FAIM appears to affect insulin signalling by regulating the expression of IR*β* and IRS and activation of Akt. These findings may have implications in the prevention and treatment of obesity, insulin resistance and related metabolic disorders.

## Materials and Methods

### Mouse maintenance and experiments

All procedures and experiments with mice were performed according to guidelines from the National Advisory Committee on Laboratory Animal Research. WT and FAIM-KO mice^22^ were housed under a 12-h light–dark cycle and given normal chow (4% of crude fat, 11% calories from fat, #1320 mod., Altromin, Large, Germany).

### Mouse fat/lean composition analysis

Body composition of age-matched mutant and control littermates was measured using EchoMRI-100 (Echo Medical Systems, Houston, TX, USA).^40^

### Metabolic cages

WT and FAIM-KO male mice were housed under controlled temperature and lighting with free access to food and water. At 14 weeks of age under NCD feeding, mouse was placed individually in metabolic cages for 2 days for adaptation. Food/water intake, energy expenditure, RER and physical activity were then recorded over a period of 3 days (TSE Systems, Chesterfield, MO, USA). Data were analysed by Student's *t*-test.^41^

### Mouse metabolic measurements

Mouse GTT were performed after 16 h fasting. After baseline glucose values were obtained using DiabeCHECK (Jitron, Singapore, Singapore), each mouse was given 2.0 mg glucose per gram of body weight intraperitoneally. Plasma glucose was subsequently monitored at 15, 30, 60, 90 and 120 min after the injection.

Serum glucose, free fatty acid, triglyceride, total cholesterol and insulin concentrations were measured with Glucose Colorimetric Assay Kit (Cayman Chemical, Ann Arbor, MI, USA), Free Fatty Acid Quantification Colorimetric/Fluorometric Kit (BioVision, Milpitas, CA, USA), Triglyceride Colorimetric Assay Kit (Cayman Chemical), Cholesterol Quantitation Kit (Sigma-Aldrich, St. Louis, MO, USA) and Ultra Sensitive Mouse Insulin ELISA Kit (CRYSTAL CHEM, Downers Grove, IL, USA), respectively.

### Histology and immunohistochemistry

Mouse liver tissue, epididymal adipose and brown adipose were fixed in 10% neutral-buffered formalin, embedded in paraffin and sectioned at 5 *μ*m. H&E staining was performed using standard techniques.

Pancreatic tissues were harvested, embedded in Tissue-Tek, and ‘snapfrozen' in dry ice and ethanol and stored at −80 °C. Cryostat sections (10 *μ*m in thickness) were prepared, air-dried and fixed in ice-cold acetone for 15 min. Sections were blocked with 5% goat serum, stained with anti-Insulin antibody (Santa Cruz Biotechnology, Dallas, TX, USA), mounted with histofluid mounting medium (Paul Marienfeld GmbH & Co. KG, Lauda-Königshofen, Germany) and analysed with an Olympus FV1000 microscope (Olympus, Shinjuku-ku, Tokyo, Japan). Images were acquired with Olympus Fluoview Version 2.1 software.

### Isolation, culture and treatment of hepatocytes

Mouse hepatocytes were isolated by two-step perfusion procedure using collagenase H according to the manufacturer's protocol (Roche Diagnostics, Basel, Switzerland). Isolated hepatocytes were plated at 3.0 × 10^5^ cells/ml density in gelatin-coated six-well plates (Becton Dickinson) in DMEM culture medium (1.1 g/l glucose) (Life Technologies, Rockville, MD, USA) containing MEM vitamins 1 × MEM amino acid 1 × nonessential amino acid 1 × 2 mM glutamine, 1% lactate (pH 7.4), 1% penicillin/streptomycin, 5% fetal calf serum and maintained at 37 °C under atmosphere of 5% CO_2_, 95% air.

### Islet isolation and treatment

Mouse pancreatic islets were extracted using a protocol modified from Bowen *et al.*^42^ by infusing common bile duct with collagenase P (Roche Diagnostics) and hand-picking the islets.^43^ After isolation, islets of wild-type and FAIM-deficient males were aliquoted at 10 islets per well in 48-well plates and cultured for 2 h at 37 °C in RPMI 1640 containing 10% heat-inactivated fetal calf serum, 10 mM glucose, 100 IU/ml penicillin and 100 μg/ml streptomycin. After brief centrifugation, 2 ml of the same medium was replaced. The clusters were then cultured for 2 h. Culture medium was collected and subjected to determination of insulin concentration.

### Immunoblot analysis

Mouse liver, skeletal muscle or white adipose tissue were dissected and immediately frozen in liquid nitrogen. Whole-cell extracts were prepared using lysis buffer (10 mM Tris-HCl, pH 8.0, 150 mM NaCl, 1 mM EDTA, 1% Igepal CA-630, 0.2mM Na_3_VO_4_ and a protease inhibitor cocktail (Roche Diagnostics)). Protein concentration was measured by colorimetric assay (Bio-Rad Laboratories, Richmond, CA, USA) and equal amount of proteins was loaded onto SDS gels. After transfer to polyvinylidene difluoride membranes, proteins were probed with primary antibodies (1 *μ*g/ml), followed by horseradish peroxidase-conjugated secondary antibodies, washed and visualized with SuperSignal West Pico/Dura chemiluminescent substrate (Pierce-Thermo Fisher Scientific, Waltham, MA, USA). Blots were reprobed with *β*-actin-specific antibody for loading controls.

Anti-IRS-1 (Santa Cruz Biotechnology), anti-IRS-2 (Santa Cruz Biotechnology), anti-SCD-1 (Santa Cruz Biotechnology), anti-IR*β* (Santa Cruz Biotechnology), anti-pAkt2 (Cell Signaling Technology, Danvers, MA, USA), anti-SREBP-1 (Santa Cruz Biotechnology), anti-ACC*α* (Santa Cruz Biotechnology), anti-FAS (Santa Cruz Biotechnology), anti-SREBP-2 (Santa Cruz Biotechnology), anti-HMGCR (Santa Cruz Biotechnology), anti-Albumin (Santa Cruz Biotechnology) and anti-*β*-actin (Santa Cruz Biotechnology) antibodies were used as primary antibodies.

### Mouse mRNA expression analysis by qPCR

Total RNA was extracted from liver tissue using Trizol (Life Technologies) and reverse transcribed into cDNA using RevertAid (Thermo Scientific, Lafayette, CO, USA). The primer pairs used for quantitative real-time PCR are listed in Supplementary Table S2. Amplification was performed by SYBR Green Real-Time PCR Master Mixes (Life Technologies) as follows: 95 °C for 20 s and 40 cycles at 95 °C for 3 s and 60 °C for 30 s. The cycle number at which the emission intensity of the sample rose above baseline was referred to as the Ct value (threshold cycle) and was proportional to target concentration. Data presented are the average of three independent experiments.

### Lipid extraction from mouse liver and LC-MS

Mouse liver tissue was harvested, flash frozen in liquid nitrogen and stored at −80 °C prior to extraction. Subsequently, 50 mg of tissue was homogenized at 4 °C and metabolites extracted as described previously^44^ using a mixture of cold methanol, tricine and chloroform. The lipid fraction (chloroform layer) was then collected and stored at −80 °C. Prior to analysis, the lipid extract was dried using N_2_ gas and reconstituted in solvents used for LC (5:2:2:1 v/v of isopropanol:methanol:acetonitrile:water with 0.1% ammonia solution and 0.1% acetic acid) prior to LC-MS analysis. All solvents were purchased from Thermo Fisher Scientific (Waltham, MA, USA) (Optima grade) except ammonia solution (25% solution, VWR Chemicals, Briare, France) and acetic acid (Merck Millipore, Darmstadt, Germany).

The reconstituted extracts were analysed in replicates using a UPLC system (Acquity, Waters, Milford, MA, USA) coupled to a QToF mass spectrometer (Xevo G2 QToF; Waters) using a reversed phase (C18) LC column (Acquity CSH, 2.1 × 50  mm, 1.7 *μ*m; Waters). The LC-MS data preprocessing and statistical analyses were performed as previously described.^45^ Briefly, a combination of univariate (Student's *t*-test with Welch correction) and multivariate (principal component analysis and orthogonal projection to latent structures-discriminant analysis) statistical tools were used to identify key differential mass peaks between wildtype and FAIM^−/−^ liver samples. Mass peaks were first putatively identified based on mass comparison (<5 ppm error) with entries from Kyoto Encyclopedia of Genes and Genome (www.genome.jp/kegg) and Human Metabolome Database (www.hmdb.ca). Subsequently, the identities of shortlisted mass peaks of interest were confirmed by MS-MS spectral comparison with commercially available standards where possible, or by comparison to an online mass spectral database (www.massbank.jp).

### LC-MS

The LC method utilized a reversed phase (C18) LC column (Acquity CSH, 2.1 × 50 mm, 1.7 *μ*m; Waters) with two solvents: ‘A' consisted of a mixture of acentonitrile, methanol and water (2:2:1) with 0.1% acetic acid and 0.1% ammonia solution, while ‘B' consisted of isopropanol with 0.1% acetic acid and 0.1% ammonia solution. Samples were run according to the following LC program: the column was first equilibrated for 1 min at 1% B with a flow rate of 0.4 ml/min. The gradient was then increased from 1% B to 82.5% B over 7.5 min before B was increased to 99% for a 3.5 min wash (flow rate for wash step is 0.5 ml/min). The column was re-equilibrated for 1 min at 1% B. The column temperature was maintained at 45 ºC. The eluent from the UPLC system was directed into the MS. Electrospray ionization (ESI) in the MS was conducted in both positive and negative modes in full scan with a mass range of 50–1200*m*/*z*. The source temperature and desolvation temperature was set at 120  and 600 °C, respectively, while the cone gas flow and desolvation gas flow were fixed at 50  and 750 l/h, respectively. The lock mass compound was leucine enkephalin (*m*/*z* 556.2771). The capillary voltage was 2 kV for the positive ESI mode and 1 kV for the negative ESI mode.

## Abbreviations

BMI: body mass index
FAIM: Fas apoptosis inhibitory molecule
FAIM-KO: FAIM deficient
FAS: fatty acid synthase
GTT: glucose tolerance test
HOMA-IR: homoeostatic model assessment of insulin resistance
HMGCR: 3-hydroxy-3-methyl-glutaryl-CoA reductase
IRβ: insulin receptor beta
IRS-1: insulin receptor substrate-1
IRS-2: insulin receptor substrate 2
LDLR: low-density lipoprotein receptor
LC-MS: liquid chromatography-mass spectrometry
MRI: magnetic resonance imaging
NCD: normal chow diet
qRT-PCR: quantitative real-time PCR
SCD-1: stearoyl-CoA desaturase 1
SREBP: sterol regulatory element-binding protein
WT: wild type
XIAP: X-linked inhibitor of apoptosis protein
