# Supplementary figures and images for: Loss of Fas apoptosis inhibitory molecule leads to spontaneous obesity and hepatosteatosis
Source: Cell Death Dis. 2016 Feb 11;7(2):e2091–. doi: 10.1038/cddis.2016.12 (PMC4849152; doi:10.1038/cddis.2016.12)

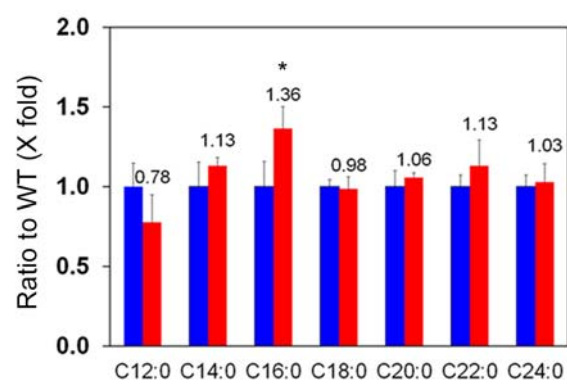

Supplement: Supplementary Figure 1 [file cddis201612x1.pdf]

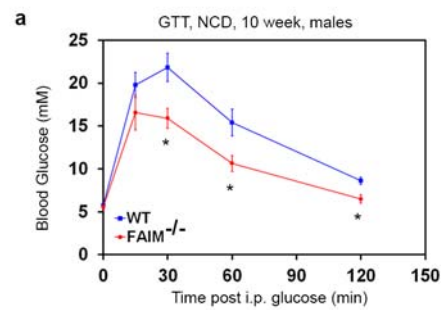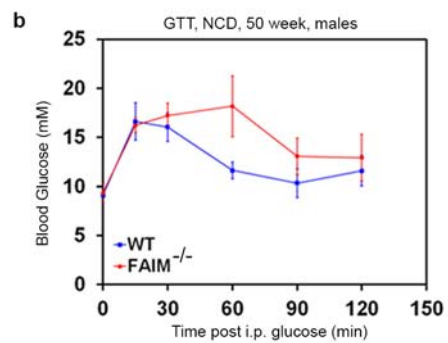

Supplement: Supplementary Figure 2 [file cddis201612x2.pdf]

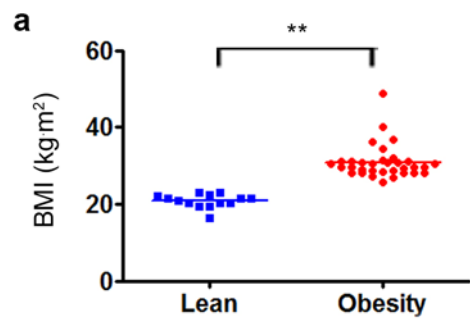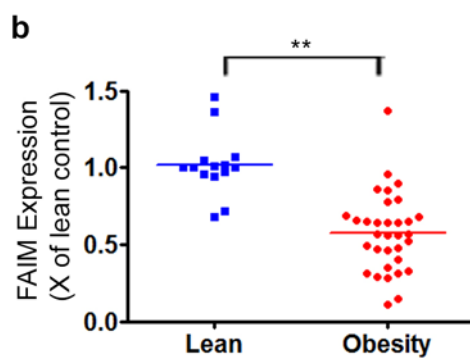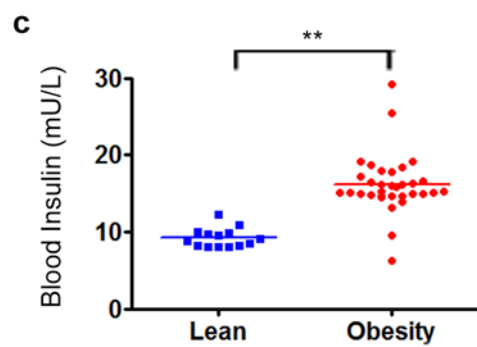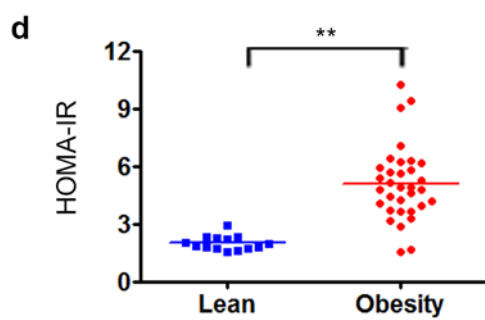

Supplement: Supplementary Figure 3 [file cddis201612x3.pdf]

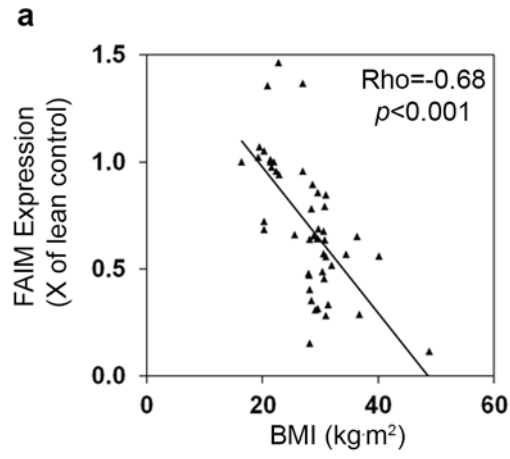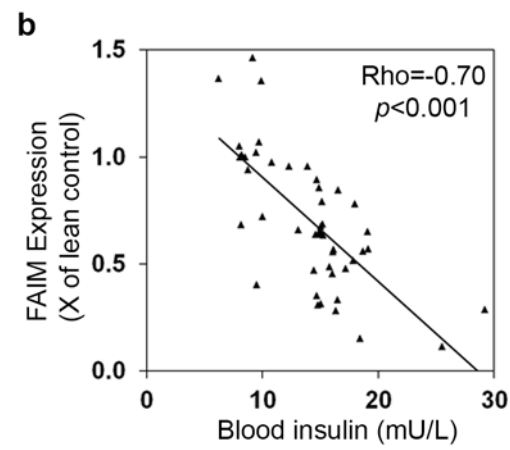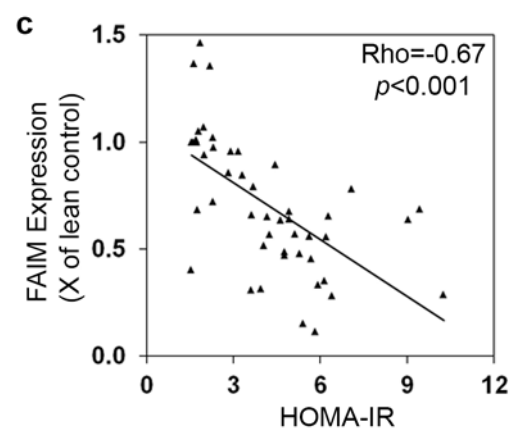

Supplement: Supplementary Figure 4 [file cddis201612x4.pdf]
