# Supplementary material for: Loss of Fas apoptosis inhibitory molecule leads to spontaneous obesity and hepatosteatosis
Source: Cell Death Dis. 2016 Feb 11;7(2):e2091–. doi: 10.1038/cddis.2016.12 (PMC4849152; doi:10.1038/cddis.2016.12)
Supplement: Supplementary Table 1 [file cddis201612x5.pdf]

**Supplementary Table 1. Association of FAIM expression with clinical and biochemical variables in multivariable linear regression model.**

| Variables | Unstandardized coefficients | Standardized coefficients | *p* value |
|---|---|---|---|
| **Age (years)** | -0.003 | -0.187 | 0.102 |
| **Gender** | -0.123 | -0.198 | 0.071 |
| **BMI (kg/m$^2$)** | -0.025 | -0.477 | 0.002 |
| **HOMA-IR** | -0.049 | -0.337 | 0.019 |
| **Triglycerides (mM)** | 0.035 | 0.148 | 0.205 |
| **HDL-Cholestrol (mM)** | 0.089 | 0.082 | 0.477 |
| **LDL-Cholestrol (mM)** | 0.058 | 0.152 | 0.284 |
