# Supplementary material for: Loss of Fas apoptosis inhibitory molecule leads to spontaneous obesity and hepatosteatosis
Source: Cell Death Dis. 2016 Feb 11;7(2):e2091–. doi: 10.1038/cddis.2016.12 (PMC4849152; doi:10.1038/cddis.2016.12)
Supplement: Supplementary Table 2 [file cddis201612x6.pdf]

Supplementary Table 2. RT-PCR primers applied in the study

Gene	Sequences of forward and reverse primers	Ref.
GAPDH	TGTGTCCGTCGTGGATCTGA	46
	CCTGCTTCACCACCTTCTTGAT	
SREBP-1a	GGCCGAGATGTGCGAACT	46
	TTGTTGATGAGCTGGAGCATGT	
SREBP-1c	GGAGCCATGGATTGCACATT	46
	GGCCCGGGAAGTCACTGT	
SREBP-2	GCGTTCTGGAGACCATGGA	46
	ACAAAGTTGCTCTGAAAACAAATCA	
HMG CoA synthase	GCCGTGAACTGGGTCGAA	46
	GCATATATAGCAATGTCTCCTGCAA	
HMG CoA reductase	CTTGTGGAATGCCTTGTGATTG	46
	AGCCGAAGCAGCACATGAT	
Farnesyl diphosphate synthase	ATGGAGATGGGCGAGTTCTTC	46
	CCGACCTTTCCCGTCACA	
Squalene synthase	CCAACTCAATGGGTCTGTTCCT	46
	TGGCTTAGCAAAGTCTTCCAACT	
LDL receptor	AGGCTGTGGGCTCCATAGG	46
	TGCGGTCCAGGGTCATCT	
Acetyl CoA carboxylase	TGACAGACTGATCGCAGAGAAAG	46
	TGGAGAGCCCCACACACA	
Fatty acid synthase	GCTGCGGAAACTTCAGGAAAT	46
	AGAGACGTGTCACTCCTGGACTT	
Stearoyl CoA desaturase-1	CCGGAGACCCCTTAGATCGA	46
	TAGCCTGTAAAAGATTTCTGCAAACC	
ApoB	CGTGGGCTCCAGCATTCTA	46
	TCACCAGTCATTTCTGCCTTTG	
ApoE	GCTGGGTGCAGACGCTTT	46
	TGCCGTCAGTTCTTGTGTGACT	
FAIM	GCTATGGACGTATGGTGCAATGGT	NM_001033030
	ACAGTCATGGTTCCCGATACTGAAG	
β-Actin	ACGTGGACATCCGCAAAG	M28424.1
	GACTCGTCATACTCCTGCTTG	
